# Asiatic acid cyclodextrin inclusion micro-cocrystal for insoluble drug delivery and acute lung injury therapy enhancement

**DOI:** 10.1186/s12951-024-02387-7

**Published:** 2024-03-17

**Authors:** Huan Shen, Li Pan, Keke Ning, Yuefei Fang, Bahtiyor Muhitdinov, Ergang Liu, Yongzhuo Huang

**Affiliations:** 1 Zhongshan Institute for Drug Discovery, Shanghai Institute of Materia Medica, Chinese Academy of Sciences, Zhongshan, 528400 China; 2 State Key Laboratory of Drug Research, Chinese Academy of Sciences, Shanghai, 201203 China; 3 School of Pharmacy, Zunyi Medical University, Zunyi, 563003 China; 4 Institute of Bioorganic Chemistry, Uzbekistan Academy of Sciences, 83 M. Ulughbek Street, Tashkent, 100125 Uzbekistan; 5 NMPA Key Laboratory for Quality Research and Evaluation of Pharmaceutical Excipients, Shanghai, 201203 China

**Keywords:** Asiatic acid, Inclusion cocrystal, Spring-and-hover model, Lung targeting delivery, Acute lung injury

## Abstract

**Background:**

Acute lung injury (ALI) is a fatal respiratory disease caused by overreactive immune reactions (e.g., SARS-CoV-2 infection), with a high mortality rate. Its treatment is often compromised by inefficient drug delivery barriers and insufficient potency of the currently used drugs. Therefore, developing a highly effective lung-targeted drug delivery strategy is a pressing clinical need.

**Results:**

In this study, the micro-sized inclusion cocrystal of asiatic acid/γ-cyclodextrin (AA/γCD, with a stoichiometry molar ratio of 2:3 and a mean size of 1.8 μm) was prepared for ALI treatment. The dissolution behavior of the AA/γCD inclusion cocrystals followed a “spring-and-hover” model, which meaned that AA/γCD could dissolve from the cocrystal in an inclusion complex form, thereby promoting a significantly improved water solubility (nine times higher than free AA). This made the cyclodextrin-based inclusion cocrystals an effective solid form for enhanced drug absorption and delivery efficiency. The biodistribution experiments demonstrated AA/γCD accumulated predominantly in the lung (C_max_ = 50 µg/g) after systemic administration due to the micron size-mediated passive targeting effect. The AA/γCD group showed an enhanced anti-inflammatory therapeutic effect, as evidenced by reduced levels of pro-inflammatory cytokines in the lung and bronchoalveolar lavage fluids (BALF). Histological examination confirmed that AA/γCD effectively inhibited inflammation reactions.

**Conclusion:**

The micro-sized inclusion cocrystals AA/γCD were successfully delivered into the lungs by pulmonary administration and had a significant therapeutic effect on ALI.

**Graphic abstract:**

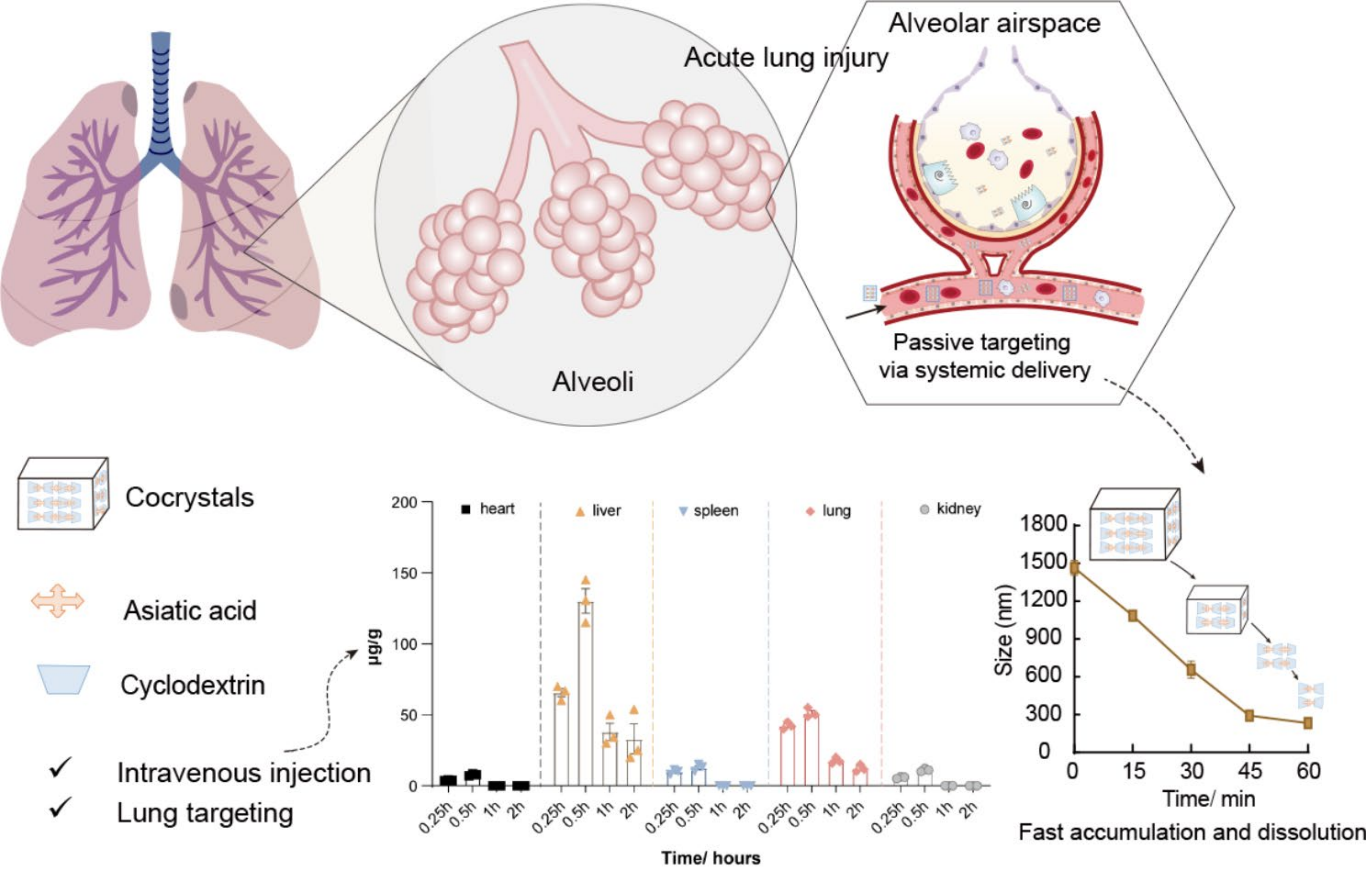

**Supplementary Information:**

The online version contains supplementary material available at 10.1186/s12951-024-02387-7.

## Background

Acute lung injury (ALI) is a fatal respiratory disease from overreactive immune reactions in response to endo- or exogenous insults, often leading to a high mortality rate and standing as one of the main causes of death in intensive care units (ICU) [1, 2]. ALI is characterized by increased lung weight on account of edema and increased pulmonary vascular permeability and is defined as the acute onset of hypoxemia [3]. Despite extensive efforts in developing therapeutic measures for ALI, the mortality of ALI remains alarmingly high (> 40%) [4, 5]. Notably, SARS-CoV-2 infection frequently precipitates ALI during the COVID-19 pandemic, and is a lead cause of death [6]. Drug delivery barriers impose a major challenge against effective ALI therapy. For example, a drug that is administered orally or by injection often cannot achieve an effective therapeutic dose in the lung [7, 8].

The size effect of a particulate form of drugs is essential for affecting the drug’s biofate. For instance, the reticuloendothelial system (RES) can sequester the particle components from circulation, usually capturing 30–90% of the injected dose [9], resulting in high accumulation in reticuloendothelium. Unlike the liver and spleen, the endothelium of the lung is lined by tight junctions that limit drugs to pass through [10], and thus the systemic distribution of the drugs to the lung is rather limited. It is reported that the average delivery efficiency for the lung is 2.8% ID/g [11]. Notably, the lung possesses the rich capillaries with a diameter of 2–3 μm, and the capillary network has a large interface (100–140 m^2^) with alveoli for gas exchange [10, 12]. With this regard, microparticles are prone to accumulated in the lung [10, 13, 14], but poorly capable of permeating the lung vasculature barrier to enter the tissue [14, 15]. Therefore, the efficient local release or dissolution is desired, and it is important that the vasculature-entrapped microparticles should disintegrate rapidly to release drug molecules to pass through lung vessel epithelium.

Crystal engineering is an elaborately technology for preparing pharmaceutical microparticles and the products are orderly packed molecular crystals with superior storage stability than the amorphous forms [16–18]. Co-crystallization is an exquisite solubilizing strategy in oral formulation development by utilizing water-soluble expedients as co-formers for hydrophobic drugs [16]. Of note, inclusion cocrystals are a specific form of cocrystals that are based host-guest inclusion between guest drug molecules and host co-formers (typically cyclodextrin). This differs from the commonly employed solubilization approach using cyclodextrin-based inclusion complexes, where the resulting product is in an amorphous state with excess cyclodextrins [19, 20]. Apart from the host-guest interaction, the formation of inclusion cocrystals involves various intermolecular forces, including hydrogen bonds and van der Waals forces [16]. Additionally, drugs and cyclodextrins within inclusion cocrystals are stoichiometrically coordinated and packed in an orderly manner, resulting in distinct crystalline structures with unique physical properties including melting point, powder density, flowability, etc. [21]. These distinct characteristics make inclusion cocrystals patentable, which is particularly valuable in the formulation-based development of new drugs. Notably, inclusion cocrystals offer a distinctive advantage over conventional cocrystals—the dissolution process of inclusion cocrystals follows a “spring-and-hover” model over the conventional cocrystals [22–24]. It means that drug/cyclodextrin host-guest complexes can be released from inclusion cocrystals efficiently and thus facilitate a fast dissolution process and maintain a high supersaturation [22, 25]. By contrast, due to fast dissolution of co-formers, rapid “parachute drop” usually happens during cocrystal dissolution, resulting in the dissolved drugs readily precipitate before absorption and decrease of drug effect [22]. Therefore, inclusion cocrystals can provide a promising pharmaceutical technology for developing microparticles for lung-targeting drug delivery.

Asiatic acid (AA) is a major triterpene isolated from *Centella Asiatica (L.) Urban* with high anti-inflammatory activity [26]. It can provide protection against hepatotoxicity [27], inhibit production of inflammatory mediators such as PGE2 and NO [28], and suppress cardiac hypertrophy by blocking NF-kB activation [29]. Furthermore, AA can attenuate lung injury by inhibiting TLR4 expression and NF-kB activation in a mouse model [30]. However, due to the unfavorable druggability (e.g., poor water solubility and low oral bioavailability), it is a big challenge in developing an efficient AA formulation. It is reported that the effective concentration of AA in vitro is in the range of 10–40 µM [26, 27, 29], corresponding to a tissue content about 5–20 µg/g for its pharmacological effect. However, the aqueous solubility of AA in water is quite poor (59.8 ng/mL at 25 °C) [31], with low oral bioavailability of 16.5% [32]. It has been analyzed that orally administrated AA at a dose of 64 mg/kg (equivalent to 10 mg/kg of in vivo administration) displayed a lung distribution of 0.5 µg/g [33], which is far below the therapeutic concentration of AA. Therefore, developing a reliable delivery strategy for sufficient lung distribution is a prerequisite for AA in the treatment of lung disease.

In this study, we designed and prepared asiatic acid/cyclodextrin inclusion cocrystals (AA/γCD) for pulmonary delivery to explore the feasibility of AA as a therapeutic agent for ALI. The dissolution behavior and mechanism, pulmonary delivery efficiency as well as the therapeutic efficacy of as-prepared micro-cocrystals AA/γCD were assessed.

## Materials and methods

### Materials

Asiatic acid (CAS: 464-92-6) was purchased from Feiyu Biotechnology Co., LTD (Nantong, China). Lipopolysaccharide (LPS, from *Escherichia coli* 0111:B4) was purchased from Sigma-Aldrich (St. Louis, USA). Methanol was purchased from Sinopharm (Shanghai, China). IL-10, IL-6, and TNF-α ELISA kits were obtained from Multisciences (Hangzhou, China). MPO kits were purchased from Elabscience (Wuhan, China). PrimeScript RT kit and SYBR Premix Ex Taq kit were purchased from YEASEN (Nanjing, China). Primers for PCR analysis were purchased from BGI (Beijing, China), with sequences shown in Table S2. γ-CD (CAS: 17465-86-0) and HPCD (CAS: 128446-35-5) were purchased from Shanghai Yuanye Bio-Technology Co., Ltd (Shanghai, China).

### Preparation of inclusion cocrystals

γCD (2.6 g) was dissolved in water to make a solution of 20 mM, and γCD solution (10 mL) was prewarmed to 50 °C and was then dropwise added into the AA methanol solution (4 mL, 2 mM) under vigorous stirring at 50 °C for 10 min. The mixture was cooled to room temperature, and further stored at 4 °C overnight for precipitation. The precipitates were collected after filtering and washing with ice alcohol, and then vacuum-dried (YB-1 A, Tianjin Xinzhou Technology, China) at 55 °C under − 0.1 MPa to obtain white powder (AA/γCD). And the drug content in cocrystals was measured by HPLC.

The HPLC method was adopted from reference [34] with minor revisions, using 0.5% acetic acid to replace 0.1% orthophosphoric acid as the water phase for a better resolution. Detailed chromatographic condition was set as follows: C18 column (250 mm × 4.6 mm, 5 μm, Waters, Milford, USA); mobile phase (0.5% acetic acid / acetonitrile, 20:80, v/v); flow rate (1 mL/min); detection wavelength (210 nm).

### Solid-state characterization

#### Physical characterization

The powder X-ray diffraction (PXRD) patterns were measured by a D/MAX 2500 X-ray diffractometer (Rigaku, Japan) with Cu Kα (0.15406 nm, 40 kV × 100 mA) as the radiation source. The samples were gently grounded and scanned with the diffraction angle (2θ) of 2°–40° (scanning rate 5°/min). The voltage and the current were set as 40 kV and 100 mA, respectively.

Fourier transform infrared (FT-IR) spectra in the range of 4000 to 400 cm^− 1^ was collected using an ATR spectrometer (Agilent, USA) with a resolution of 4 cm^− 1^ under ambient conditions.

Thermal analysis by differential scanning calorimetry and thermogravimetric analysis (TGA/DSC, STA449F3, Netzsch, Germany) were performed for measurement of melting properties and thermal stability. The measurement was carried out using 5 − 10 mg samples at a rate of 10 °C min^− 1^ under nitrogen protection.

The ^1^HNMR and 2D ROESY NMR experiments were conducted and recorded on a Bruker Avance-500 spectrometer (Bruker, Germany) at 500 MHz under ambient conditions. Chemical shifts for protons were reported in parts per million (ppm) downfield from tetramethylsilane and were referenced to residual protons in the NMR solvents (DMSO-d6, δ 2.50; D_2_O, δ 5.17).

#### Size and morphology determination

The hydrodynamic size of the samples was determined using a Nano ZS90 (Malvern, U.K.). The morphology of samples was recorded by using a microscope (Nikon, Japan), and also recorded using a scanning electron microscope (SEM) (TESCAN MIRA4, Oxford, U.K.). The SEM samples were dispersed in ethanol and attached to the SEM aluminum stubs, which were sputter-coated with a thin layer of gold before analysis.

#### Drug loading efficiency (DLE) and entrapment efficiency (EE) determination

The DLE of inclusion cocrystals were measured as follows: 10 mg of AA/γCD were weighted and dissolved by 1 mL DMSO, which were then diluted in a 100 mL-volumetric flask by methanol. Before HPLC analysis, the solutions were filtered by PVDF membrane (0.45 μm, Millipore, USA), and 20 µL sample was injected for AA determination. And the HPLC method for analysis was the same as Sect. 2.2.

The DLE% was calculated as follows:

$${DLE (\%)} = \frac{Drug content in cocrytsals}{Total solid mass}\times 100$$

The EE% was calculated using the following formula:

$${EE (\%)} = \frac{Drug content in cocrytsals}{Total drug added} \times 100$$

### Phase solubility assay

A serial γCD water solution (10 mL) of varying concentrations was prepared in the vials. Excess AA powders were added into the vials, which were stirred at 150 rpm for 24 h to achieve solubilization equilibrium at 25 °C. The suspension was then centrifuged at 8,000 rpm for 10 min, and the supernatant was collected for HPLC assay (1260 Infinity II, Agilent, Palo Alto, USA). The HPLC method for analysis was the same as Sect. 2.2. The phase solubility curve was obtained by plotting the molar concentration of solubilized AA against the initial concentration of γCD.

#### In vitro release test

In the release experiments, both the AA-HPCD inclusion complex and the AA/γCD inclusion cocrystal contained an equivalent amount of AA (2 mg). Five milliliters of AA-HPCD inclusion complex solution or 17.4 mg AA/γCD cocrystals were placed in dialysis bags (MW cutoff: 35 kDa), which were put in 200 mL PBS (pH 7.4) containing 1% Tween-80 (w/w) under constant stirring at 150 rpm. At predetermined time points (0.5 h, 1 h, 2 h, 4 h, 6 h, 9 h, 12 h, 24 h), a portion of the release medium (1 mL) was withdrawn and replenished by equivalent amounts of fresh medium. The concentrations of AA were quantified by HPLC and the accumulative drug release was calculated. The HPLC method used for analysis was the same as Sect. 2.2.

### Animal experiments

#### LPS-induced acute lung injury and treatment procedure

BALB/c mice (male, 6–8 weeks old and weighing 18–22 g) were randomly divided into four groups (*n* = 8): as Control, Model, AA-HPCD inclusion complex, and AA/γCD inclusion cocrystals. Control group received an endotracheal drip of saline, while other groups were intratracheally given with an equivalent volume of LPS (dissolved in saline), followed by intravenously administration of saline (Model group), AA-HPCD inclusion complexes (10 mg/kg), or AA/γCD inclusion cocrystals (10 mg/kg). AA-HPCD inclusion complexes was prepared by dissolving 4 mg AA in 10% HPCD saline solution (2 mL) under constant stirring (300 rpm, 25 °C) for 10 h. All the animals were housed in a specific pathogen-free facility with free access to food and water. For induction of ALI, the mice were isoflurane anesthetized, and administrated LPS (5 mg/kg, for the rest groups) by intratracheal instillation [35, 36]. Four hours after LPS treatment, AA-HPCD or AA/γCD was suspended in 100 µL saline for intravenous (in vivo) injection at a dose of AA equivalent to a 10 mg/kg. At 24 h post LPS exposure, the mice were anesthetized to collect blood from the retro-orbital plexus, and then the mice were euthanized by AWE-M animal euthanasia apparatus (Guangzhou, China) supplied with high purity CO_2_ (100%) at a flow rate of 6 L/min for 7 min. The lungs were excised and weighed, with the trachea being washed with 0.8 mL saline to collect the alveolar lavage fluid (bronchoalveolar lavage fluid, BALF). A part of the tissues was homogenized with PBS or Trizol reagent for biological analysis, and the other part was fixed by 4% paraformaldehyde for H&E (hematoxylin and eosin) staining.

The animal study was carried according to the guidelines for the care and use of laboratory animals, and conducted under the approval and supervision of the Ethics Committee on Laboratory Animal Management of the Zhongshan Institute for Drug Discovery (Approval No. 2022-06-HYZ-07).

#### Enzyme-linked immunosorbent (ELISA) assay

The BALF was centrifuged for 10 min at 15,000 rpm at 4 °C. The supernatant was collected to determine the concentrations of TNF-α, IL-10, and IL-6 by ELISA kits following the manufacturer’s instruction. The instrument used for ELISA in our manuscript was SpectraMax M5/M5e (Molecular Devices, USA).

The tissues were homogenized using a tissue grinder (TP-24, Jieling Instrument Co., Ltd, Tianjin, China) and centrifuged for 20 min (3,000 rpm, 4 °C). The supernatant was collected, and the inflammatory markers such as TNF-α, IL10, IL-6, and myeloperoxidase (MPO) were determined by ELISA assay as described above.

#### Pathological examination of lung tissues

The paraformaldehyde-fixed tissues were subsequently subjected to alcohol dehydration, xylene clearing, and paraffin embedding, which were then cut into 5-µm sections for H&E staining. The slides were imaged using an inverted microscope (Olympus X71, Japan) for further analysis.

#### Real-time fluorescence quantitative PCR

Total RNA was extracted from the lung tissues with Trizol reagent (Invitrogen, USA). cDNAs were synthesized by SuperMix kit (TransGen Biotech, Beijing, China) using oligo-(dT) as the primer. Relative expression of inflammatory cytokines against the housekeeper β-actin was determined by Real-time qPCR on a CFXTM Real-Time Thermal cycler (Bio-Rad, Hercules, USA).

#### The organ distribution of inclusion cocrystal in mice

The organ distribution of AA inclusion cocrystals in comparison with inclusion complexes in mice was analyzed by HPLC. After intravenous administration (10 mg/kg), mice were euthanized at a specified time points (0.25 h, 0.5 h, 1 h, and 2 h) to collect the tissues and blood. The organs were homogenized by a tissue grinder and AA was extracted with ethyl acetate. The organic solvent was evaporated and the residual was dissolved by methanol for HPLC determination. The HPLC method used for analysis was the same as Sect. 2.2.

### Statistical analysis

All quantitative data are presented as the mean ± SEM. The level of significance was calculated according to two-way ANOVA.

## Results

### Identification and characterization of the inclusion cocrystals

**Fig. 1:**
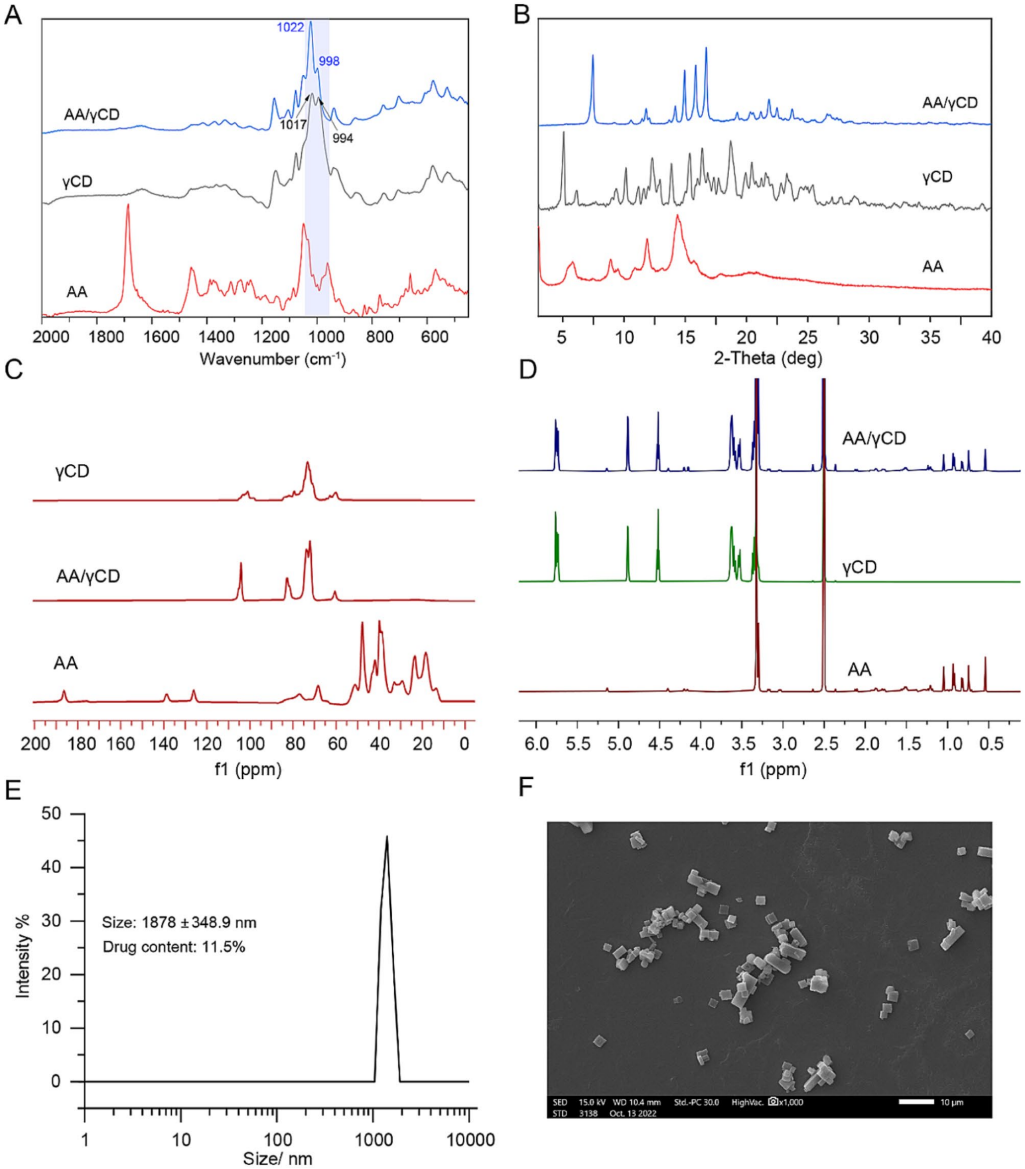
Characterization of AA/γCD cocrystals. (**A**) FTIR spectrum; (**B**) PXRD patterns; (**C**) Solid-states ^13^C-NMR; (**D**). HNMR in d_6_-DMSO; (**E**) the DLS size of cocrystals; (**F**) the SEM image of cocrystals, Scale bar: 10 μm

The AA/γCD inclusion crystals showed a cubic morphology with a hydrodynamic size of about 1.8 μm (Fig. 1E and F). The powder X-ray diffraction (PXRD) confirmed the formation of the novel crystal structure, of which the characteristic diffraction peaks (7.5, 9.2, 10.6, 11.5, 11.8, 12.1, 13.7, 14.2, 14.9, 15.8, 16.7, 19.2, 20.3, 21.2, 21.8, 22.5, 23.7, 26.6) were different from either γ-CD (Fig. 1B) or AA crystal form. H-NMR spectrum of the as-prepared crystal identified the co-existence of both AA and γCD, demonstrating the product was a cocrystal of AA and γCD with stoichiometry molar ratio of 2:3 (AA: γCD) (Fig. 1D). Further, the FTIR spectrum of AA/γCD was similar to γCD (Fig. 1A), but the characteristic peaks of 1022 cm^− 1^ and 998 cm^− 1^ (represented the -C-O-C- stretching vibration) were slightly redshifted, indicating a weak interaction between AA and γCD, such as hydrogen bond and Vanderwal’s force [27]. The characteristic peaks of AA in the cocrystal almost disappeared, suggesting the functional structures of AA was embedded by γCD. The solid ^13^C-NMR patterns showed that most of the AA characteristic peaks disappeared in the AA/γCD cocrystal was consistent with the results of FTIR spectra (Fig. 1C), suggesting AA has entered the hydrophobic cavity of γCD. The water content of AA/γCD determined by TGA was 14.6%, while the mass change attributed to water loss was 7.5% in γCD (Fig. S1). By subtracting the mass content of AA and γCD, it was calculated that about 81 water molecules were embedded in the cocrystals. The DSC result showed that the melting point of AA/γCD (about 278 °C) was different from either γCD (297 °C) or AA (325 °C). Interestingly, the endothermic peak corresponding to free water loss in AA and γCD appeared below 100 °C. In comparison, the water loss of AA/γCD appeared below 135 °C, suggesting the increased stability of cocrystals.

The DLE and EE of the inclusion cocrystal (also referred to the production yield in crystallization industry) were determined by a modified HPLC method with a linearity range of 3–200 µg/mL (calibration curve: y = 9.6058x + 3.1065, R² = 0.9995, see Fig.S2) and a detection limit of 0.75 µg /mL. By this method, DLE and EE of AA/γCD was calculated to be 11.4% and 91.2%, respectively. Further, the drug content was measured after one month of storage at room temperature, which showed no significant change in drug content (Table S1), indicating that the inclusion cocrystals had a good stability. Water solubility of AA/γCD cocrystals (about 91 µg/ml) is greatly improved, which is 9 times higher than that of free AA (< 10 µg/ml).

### Lung targeting and safety assessment of AA/γCD inclusion cocrystals

**Fig. 2:**
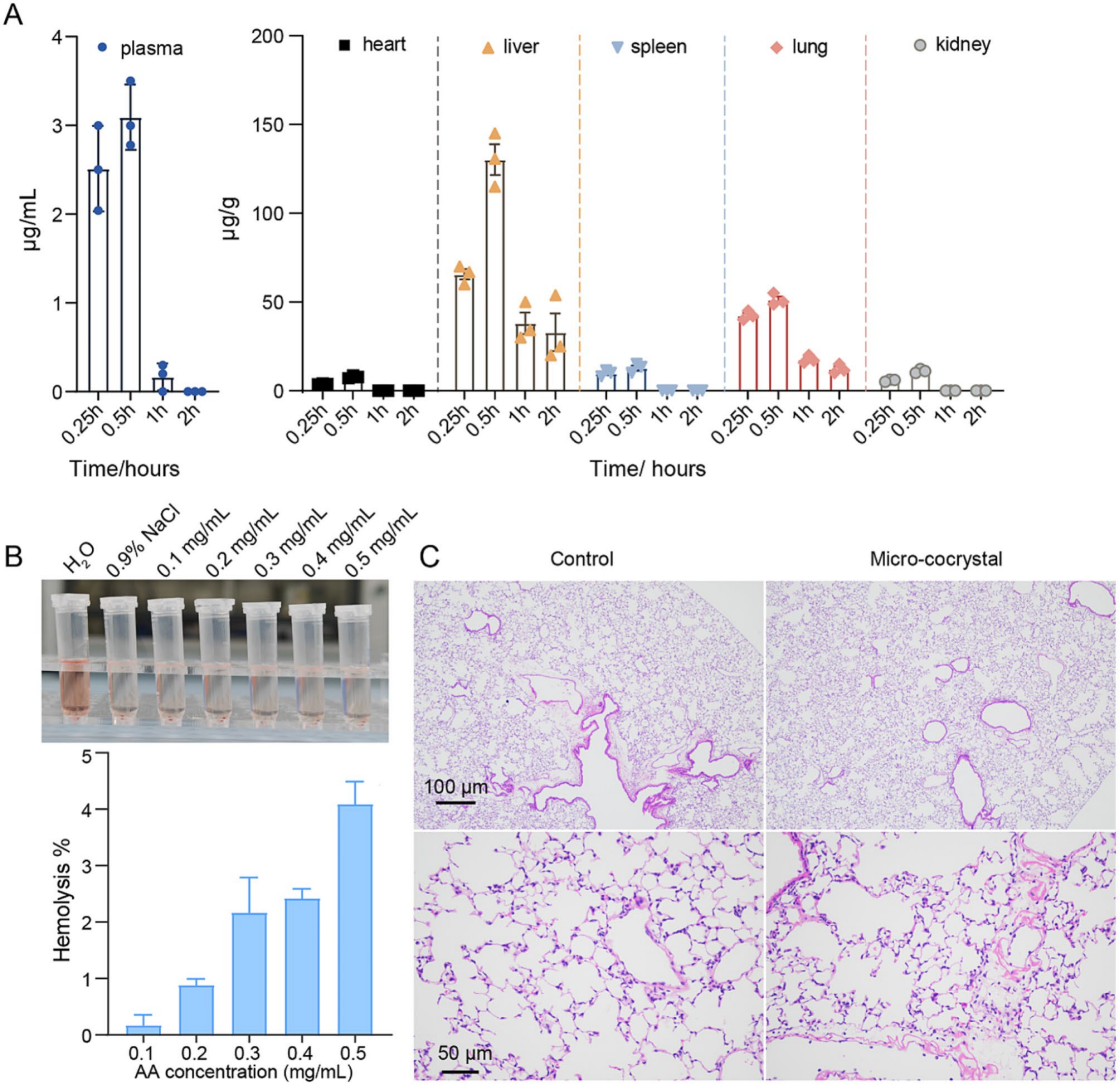
Lung targeting assessment of AA/γCD. (**A**) Organ concentration of inclusion cocrystal AA/γCD in plasma, heart, liver, spleen, lung, and kidney at 0.25 h, 0.5 h, 1 h, and 2 h after intravenous injection (in vivo). Dose: 10 mg/kg. (**B**) The hemolysis effect of AA/γCD cocrystal in 0.1–0.5 mg/mL. (**C**) HE staining of lung sections after three consecutive injections of AA/γCD cocrystals (10 mg/kg)

For ALI treatment, systemic delivery to the injured alveolar region is more efficient than usual because of increased alveolar–capillary permeability. We investigated the biodistribution and the safety of inclusion cocrystals via in vivo administration. It was shown that the AA/γCD inclusion cocrystals were fast cleared from circulation and mainly distributed into the liver and lung after in vivo injection. The drug concentration showed a peak at 0.5 h (Fig. 2A), which declined subsequently, probably due to the quick metabolism of AA (t_1/2_=9.5 min) [32]. Of note, drug concentration in the lung was significantly higher than that in the blood, heart, spleen, and the kidneys. We evaluated the biosafety of the AA/γCD cocrystals via in vitro and in vivo experiments. Hemolysis assay demonstrated that AA/γCD cocrystal did not induce significant hemolysis (Fig. 2B). In addition, after three consecutive injections of AA/γCD (10 mg/kg), H&E staining of lung sections showed no obvious damages, indicating the safety of in vivo administration of AA/γCD (Fig. 2C).

### The release kinetics of AA/γCD inclusion cocrystal

**Fig. 3:**
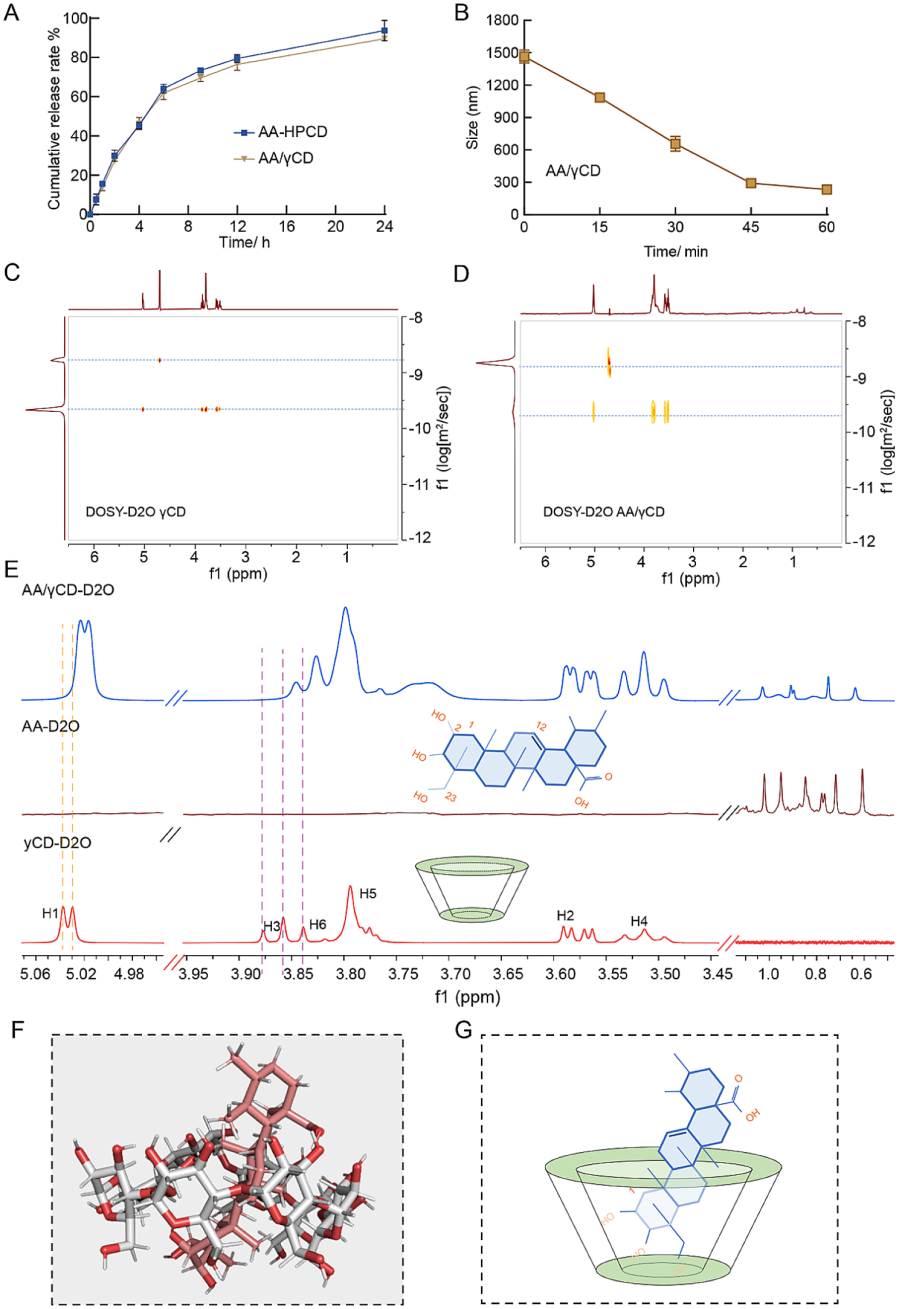
(**A**) In vitro drug release profiles of AA-HPCD inclusion complex and AA/γCD inclusion cocrystals. (**B**) DLS size changes during the release of AA/γCD cocrystals. (**C**) & (**D**) DOSY patterns of γCD, and dissolved AA/γCD cocrystals in D_2_O. (**E**) HNMR spectrums of γCD, AA, and AA/γCD cocrystal. (**F**) & (**G**) Molecular docking and simplified illustration of AA/γCD inclusion complex (Autodock vina)

Microparticles accumulated in bronchioles should disassemble fast to release free drug molecules for alveolar permeation. We investigated the release behaviors of AA/γCD cocrystals in PBS (pH 7.4) using AA-γCD inclusion complexes as a control. The fast release profile of AA/γCD cocrystals was similar to the AA-γCD inclusion complex, reaching 90% in 24 h (Fig. 3A). The particle sizes of AA/γCD cocrystals decreased from a micrometer size to 233 nm within 1 h (Fig. 3B), indicating a rapid dissolution. The freshly released drug from AA/γCD cocrystals was in an inclusion complex form, because DOSY-HNMR (Diffusion-Ordered Spectroscopy) showed an only peak of inclusion complex (Fig. 3C and D), and no HNMR signals of the dissociated guest molecules were detected. This release behavior thus could enhance the solubility of the released drug by maintaining an inclusion complex from, and avoided heterogeneous growth of particles because of *Vilhelms Ostvalds* mechanism. In addition, the conformation of the guest molecule in a γCD cavity was deduced as shown in Fig. 3F, according to the chemical shift of H-NMR of free AA, γCD, and AA/γCD in D_2_O (Fig. 3E). The docked simulation of the inclusion complexes revealed the lowest energy conformation where hydroxyl groups of the inclusive AA was adjacent to the primary side of CD molecule (Fig. 3G), which was consistent with the H-NMR results.

Cocrystal formation and dissolution is a dynamic and reverse process. The phase solubility of AA was tested in the γCD solutions with varying concentrations. The determined AA in the solution were plotted against the γCD concentration, producing the aqueous solubility profile (Fig. S3A). As shown, the solubility curve of AA was composed of three equilibrium stages: At stage I (C_γCD_ ≤ 1.8 mM) and stage II (1.8 mM < C_γCD_ ≤ 7.2 mM), AA solubility rose linearly in response to the increasing C_γCD_ with different slopes (0.80 for stage I, and 0.18 for stage II) (Fig. S4). Since the soluble form of AA were maintained by γCD, decreased slope of stage II curve suggested the formation of higher-molar-ratio complex (γCD : AA) in the solution, which coincided with the increased DLS size of stage II than stage I (Fig. S3B). When C_γCD_ was above 7.2 mM (stage III), soluble AA concentration began to decrease in ascending concentrations of γCD (Fig. S3A) and the veritable C_γCD_ in solution was declined sharply (Fig. S5), suggesting the complexes of AA and γCD began to precipitate from solution. In line with this, PXRD spectrum of the precipitates began to transform into AA/γCD inclusion cocrystals (the main characteristic diffraction peaks: 7.5, 11.8, 12.1, 14.2, 14.9, 15.8, 16.7, 19.2, 20.3, 21.2, 21.8, 22.5, 23.7, 26.6) from the feeding AA powders (the main characteristic diffraction peaks: 5.8, 7.4, 8.9, 9.5, 11.9, 14.3, 15.8, 17.9, 20.3) since stage II (Fig. S3C). The C_γCD_-dependent phase solubility change and cocrystal formation were mediated by the equilibrium between the undissolved AA, soluble AA molecules and γCD molecules, AA-γCD complexes, and the inclusion cocrystals, thereby increased γCD amount could promote the formation of AA-γCD complexes, and the production of γCD/AA cocrystals (Fig. S3D). In this account, the dissolution of AA/γCD inclusion cocrystals would fast release the AA-γCD complexes, thus displaying a similar release profile with AA-HPCD complexes.

### In vivo therapeutic effect of AA/γCD inclusion cocrystals on LPS-induced ALI

**Fig. 4:**
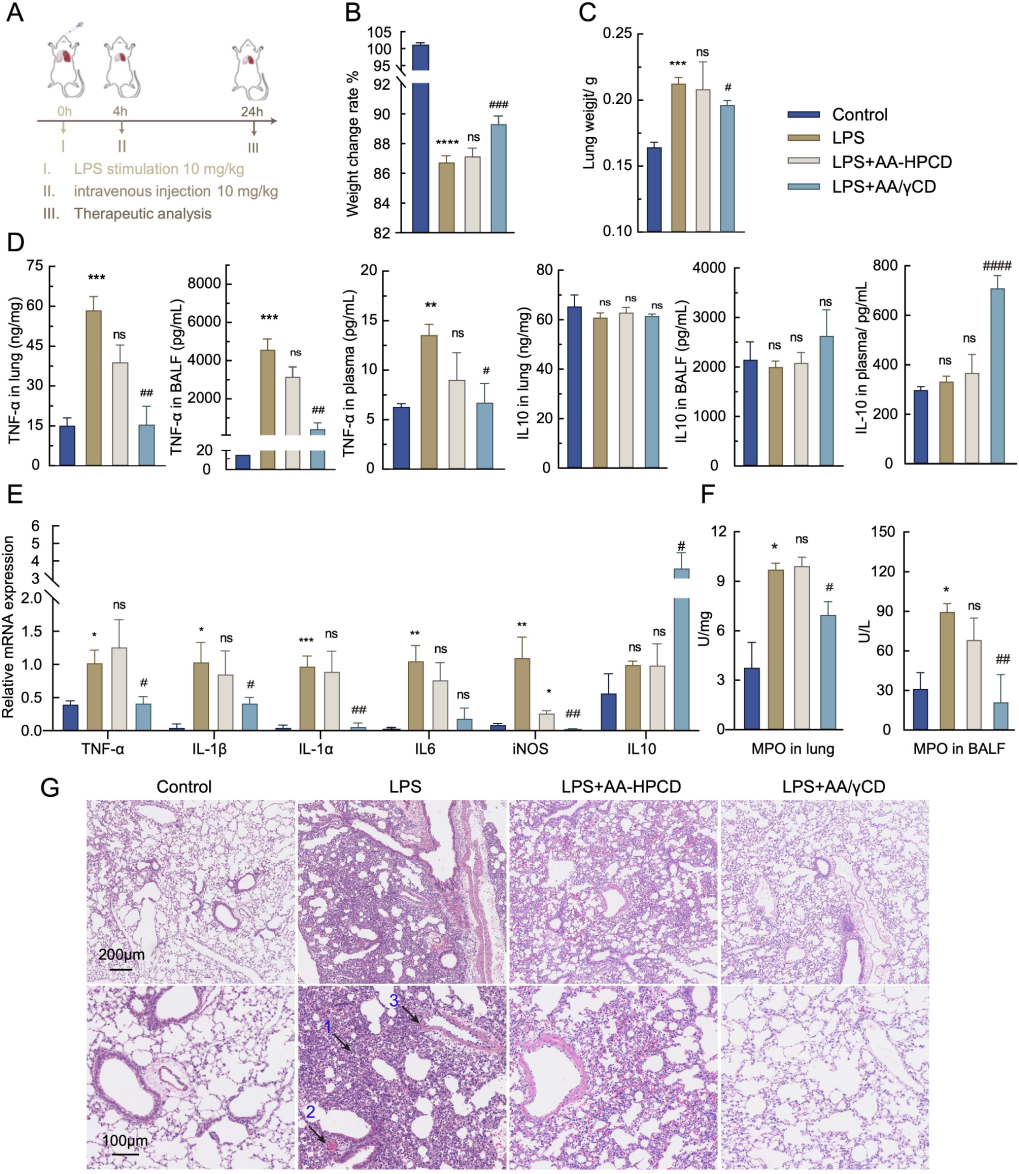
In vivo therapeutic assessment of AA/γCD in treatment of mice ALI. (**A**) Experiment schedule. LPS (10 mg/kg) was intratracheally injected to induce ALI, and AA/γCD was intravenously administered 4 h later. Normal mice exposed to saline challenge served as the control. (**B**) Percentage of body-weight change of mice at 24 h after LPS treatment. (**C**) Lung index. (**D**) MPO activity of lung homogenates and BALF. (**E**) TNF-α and IL10 in lung homogenates, BALF, and plasma were assessed by ELISA. (**F**) mRNA levels of TNF-α, IL-1α, IL-1β, IL-6, IL-10 in lung homogenates determined by real-time qPCR. (**G**) H&E-stained lung sections. Arrows indicate: 1, thickening of the alveolar and infiltration of inflammatory cells; 2, hemorrhage; 3, interstitial edema. Statistical comparison between control and other groups are marked by *, with *, **, and *** representing  < 0.05, P < 0.01, and *P < 0.001, respectively.* Between-group comparisons with LPS are marked by *#, with #, ##, and ### representing P < 0.05, P < 0.01*, and *P < 0.001, respectively*

ALI is usually initiated by acute inflammation in response to endo- and exogenous insults, leading to a series of pro-inflammatory events and increased permeability of pulmonary vasculatures [3, 37]. The immune cells are recruited to the alveoli, and ALI shows elevated inflammatory factors (IL-6, IL-8, TNF-α) with high mortality [5]. To this account, inflammation control to prevent immune damage is critical for ALI treatment. We evaluated the therapeutic efficacy of AA/γCD cocrystals on LPS-induced ALI of mice using the solution of AA-HPCD inclusion complexes as control (Fig. 4A). Intratracheal instillation of LPS caused significant weight loss (14%) and lung edema with an increased lung index from 0.16 to 0.21 (Fig. 4B and C). Meanwhile, TNF-α was significantly increased in both the lung and bronchoalveolar lavage fluids (BALF) (Fig. 4E). The qPCR results showed that the inflammatory factors including TNF-α, IL-1β, IL-1α, IL-6, and iNOS were all significantly elevated in the LPS-treated mice (Fig. 4F), but these factors were significantly downregulated after in vivo administration of AA/γCD cocrystals. Of note, myeloperoxidase (MPO) was an enzyme located mainly in the primary granules of neutrophils, serving as an indicator of inflammation. AA/γCD treatment significantly decreased MPO in the lung tissues (about 25%) and the BALF (about 65%), suggesting the significantly ameliorated neutrophil infiltration into the lung parenchyma and alveolar spaces (Fig. 4D). In addition, IL-10 levels in the lung tissues, BALF, and plasma were increased after AA/γCD treatment, suggesting that AA/γCD cocrystal exerted anti-inflammatory effects. H&E staining also demonstrated that the AA/γCD cocrystal could notably alleviate LPS-induced tissue damages (e.g., interstitial edema, hemorrhage, and thickening of the alveolar wall) (Fig. 4G). In comparison, the inclusion complexes of AA-HPCD had inferior therapeutic efficacy on ALI. The results indicated that inclusion cocrystals could benefit from the micro-size effect and yield enhanced pulmonary drug accumulation.

## Discussion

The phenomenon of preferential accumulation of particles in the lungs due to the micrometer-scale dimensions of pulmonary vasculature is well-documented [10, 13]. Of note, the sufficient local release from the accumulated microparticles in the lung would benefit free drug molecules permeate through the vascular endothelium. The merit of this work lies in providing an efficient lung delivery strategy using inclusion micro-cocrystal via size effect-based passive targeting mechanism. The developed inclusion cocrystals are distinct from typical cocrystals utilizing small molecular conformers such as organic acids and amino acids. Inclusion cocrystals also differs from inclusion complexes in its fixed chemical stoichiometry, ordered crystal structure, and unique physicochemical properties. Importantly, inclusion cocrystals are patentable, thus with potential value in translation.

**Fig. 5:**
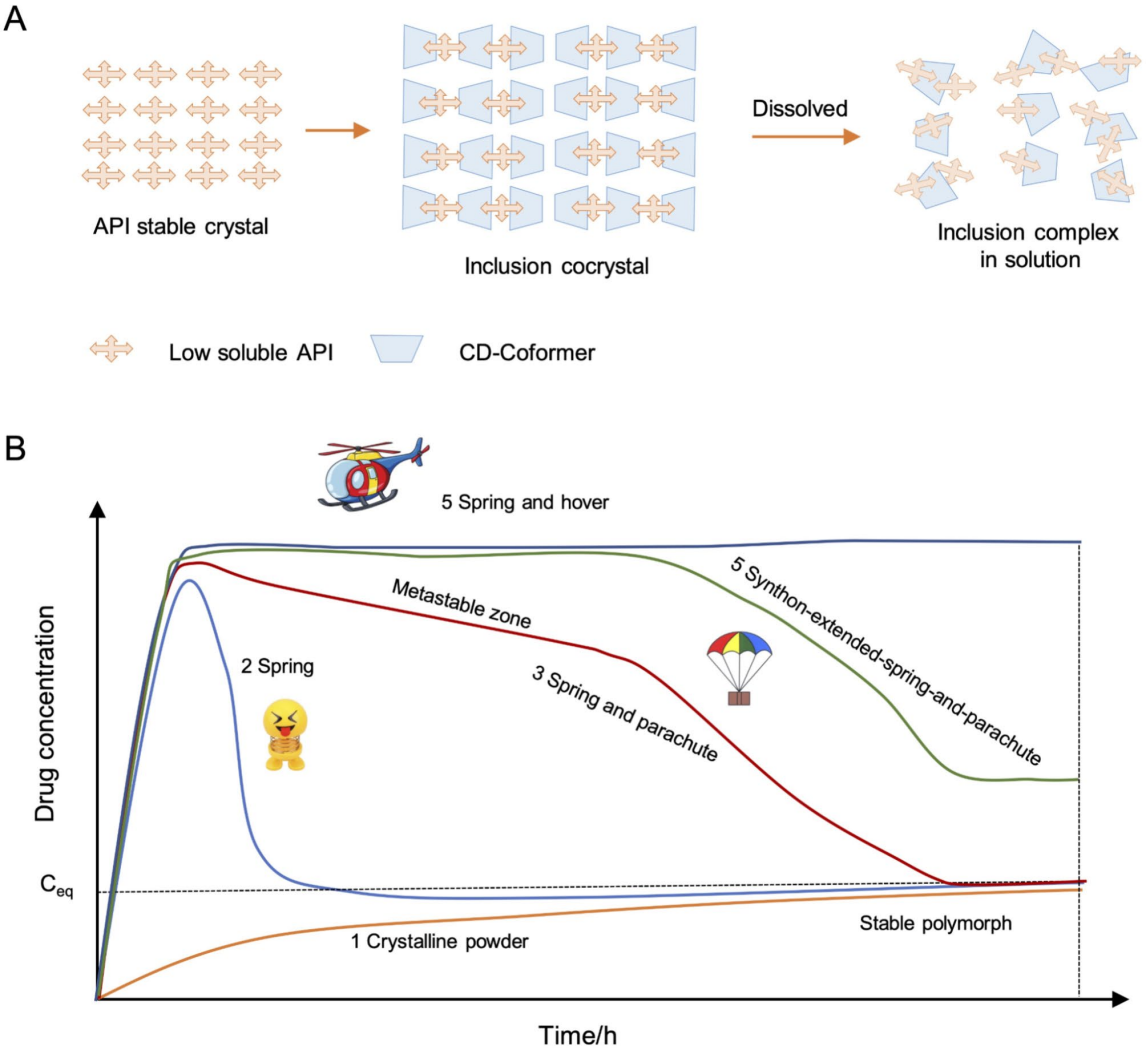
(**A**) Illustrated dissolution process of AA/γCD cocrystals. (**B**) Five typical dissolution profiles of API. (1) Dissolution of insoluble drug with stable crystalline form; (2) Insoluble drug in metastable forms (i.e., amorphous phase) shows quick dissolution and achieves the peak concentration which quickly drops (within minutes to an hour) and transforms to an insoluble crystalline form. (3) Cocrystals can dissolve fast and the supersaturated concentration can be maintained for hours (metastable zone) because of the assistance of water-soluble coformers. (4) Insoluble drugs dispersed by pharmaceutic surfactants can fast disintegrate, and the released API can be stabilized by the surfactant, thus effectively extending the life of the API in solution. (5) Highly soluble drugs can quickly dissolve and maintained a constant concentration in the media

As a great advantage, the dissolution behavior of AA/γCD inclusion cocrystals follows a “spring-and-hover” model, and AA can dissolve and release in a form of AA inclusion complex (Fig. 5A). Generally, there are five typical dissolution profiles for solid-state molecules (Fig. 5B). (1) Most crystalline powders dissolve slowly and readily reach a plateau when saturation solubility is achieved. (2) Amorphous powders can dissolve fast and readily reach a supersaturation status. But the solute crystallization happens simultaneously, and the solubilized drug concentration will immediately drop when the amorphous powders are exhausted, showing a “spring” dissolution profile, which is unfavored for drug absorption [38]. (3) To improve the dissolution, cocrystals are explored in recent years [39]. Cocrystals can take advantage of solubility-enhancing co-formers that promote dissolution and maintain a supersaturation status of the solubilized drugs [22, 24, 40]. However, the solubility-enhancing effect of co-formers is concentration-dependent. If co-formers are diluted by body fluids (e.g., gastrointestinal fluids or blood), and thus lead to a “fast parachute” phenomenon and the consequent decrease in dissolution and bioavailability. (4) By optimizing the crystalline co-formers, it is expected to achieve synthon-extended-spring and parachute dissolution [23], which, however, usually needs high content of specialized co-formers and thus is not feasible for pharmaceutical application. (5) The dissolution of inclusion cocrystal release inclusion complexes of AA, which is stable in an aqueous solution, and thus can maintain a solubilized form, displaying a “spring-hover” profile. Therefore, the AA/γCD micro-cocrystals trapped by pulmonary capillaries after in vivo injection can rapidly dissolve and permeate into the diseased alveoli, thereby avoiding embolism related side effects.

The lung delivery of micro-crystals was achieved via a passive-targeting mechanism of size effect. Microparticles are prone to accumulate in the lung [10, 13], because the lung possesses the rich capillaries with a diameter of 2–3 μm, and the capillary network has a large interface (100–140 m^2^) with alveoli for gas exchange [10, 12]. Additionally, cocrystal microparticles in the lung vessels can rapidly dissolve and release the drug inclusion complex, facilitating drug permeation into the lungs. Therefore, the enhanced drug accumulation and release into the lung tissue leads to the improved therapeutic efficacy. Pro-inflammatory immune cells play important roles in acute lung injury, which can release abundant inflammatory cytokines to drive disease progression. This work revealed that the therapeutic action of AA/γCD micro-cocrystals involve the decrease of neutrophil infiltration into the lung and downregulation of inflammatory factors such as TNF-α, IL-1β, IL-1α, IL-6, and iNOS. Yet, further studies on detailed therapeutic mechanisms should be carried out to illustrate the action of AA on other immune cells and immune regulation.

## Conclusion

We developed a micro-sized inclusion cocrystals for treating ALI with an advantage of passive targeting the lung. The as-prepared AA/γCD was about 1.8 μm in size and the drug could dissolve and release in a form of inclusion complex of AA. The in vivo results demonstrated the in vivo administrated AA/γCD significantly alleviated the symptoms of ALI, and reduced pro-inflammatory cytokines both in the lung and BALF. The HE staining results confirmed that the inflammation reaction was inhibited by AA/γCD cocrystal treatment. In conclusion, the inclusion cocrystals AA/γCD could efficiently deliver drugs into the lung and represented a promising strategy for treatment of ALI.

### Electronic Supplementary Material

Below is the link to the electronic supplementary material.

Supplementary Material 1

## Data Availability

Data is provided within the manuscript or supplementary information files.
