# Supplementary material for: Asiatic acid cyclodextrin inclusion micro-cocrystal for insoluble drug delivery and acute lung injury therapy enhancement
Source: J Nanobiotechnology. 2024 Mar 17;22:119. doi: 10.1186/s12951-024-02387-7 (PMC10946140; doi:10.1186/s12951-024-02387-7)
Supplement: Supplementary file 1 — Supplementary Material 1 [file 12951_2024_2387_MOESM1_ESM.docx]

**Fig. S1** TGA and DSC profiles of AA, γCD, and AA/γCD inclusion cocrystals.

**
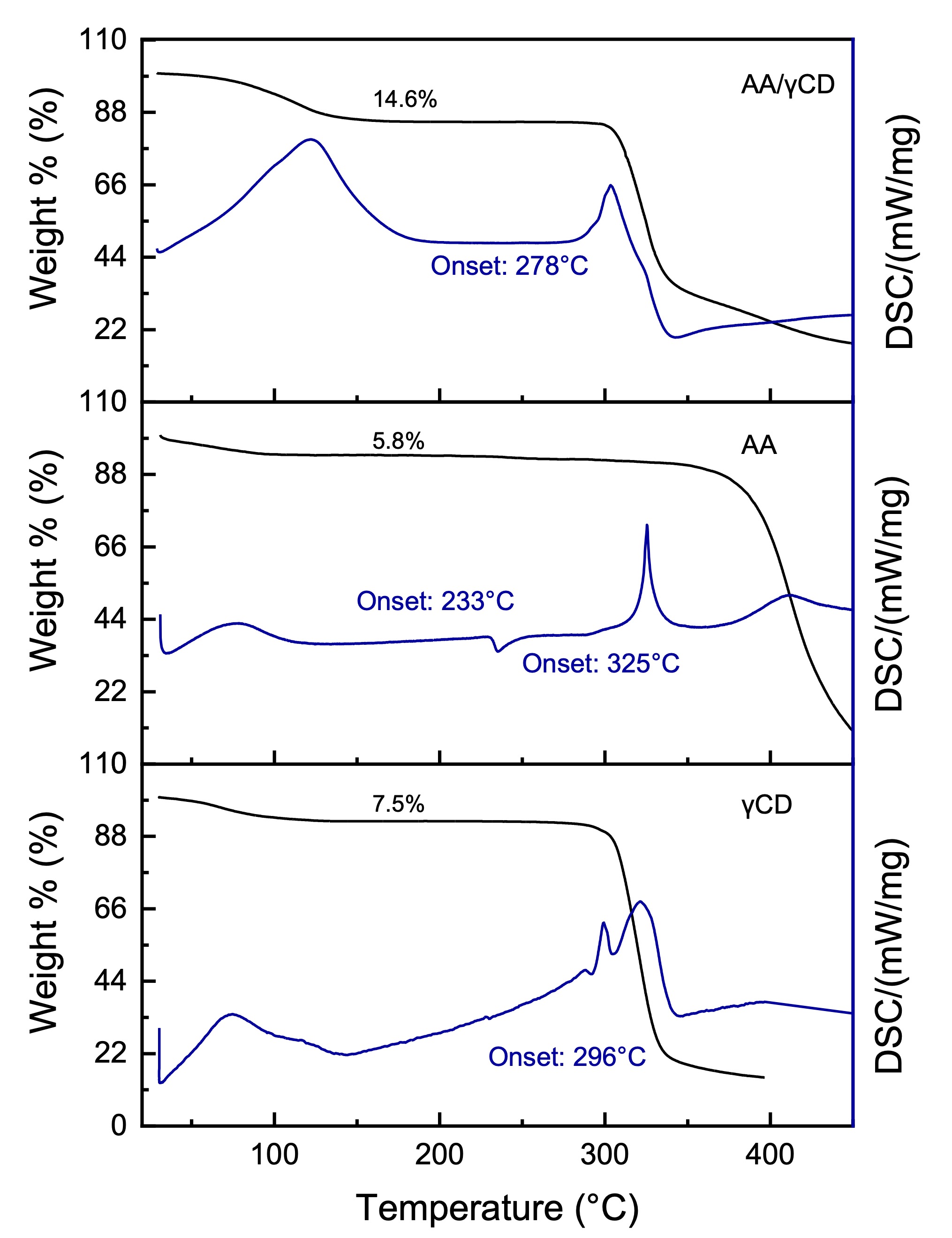
**

**Table S1** Storage stability of AA/γCD cocrystals at room temperature. AA/γCD cocrystals were stored at room temperature for 1 month. At 0 d, 15 d, and 30 d, AA content of the samples was determined by HPLC following the protocols described in section 2.3.3.

| Time/ day | 0 | 15 | 30 |
| --- | --- | --- | --- |
| AA content/ % | 11.42% | 11.56% | 11.48% |

**Fig.S2** The HPLC standard curve of AA

**Fig. S3** (A) Phase solubility of AA in ascending concentration of γCD. (B) Monitored DLS size in supernatants from phase solubility test. (C) PXRD patterns of precipitates obtained from phase solubility tests. (D) Diagram of the three stages of phase solubility test.

**
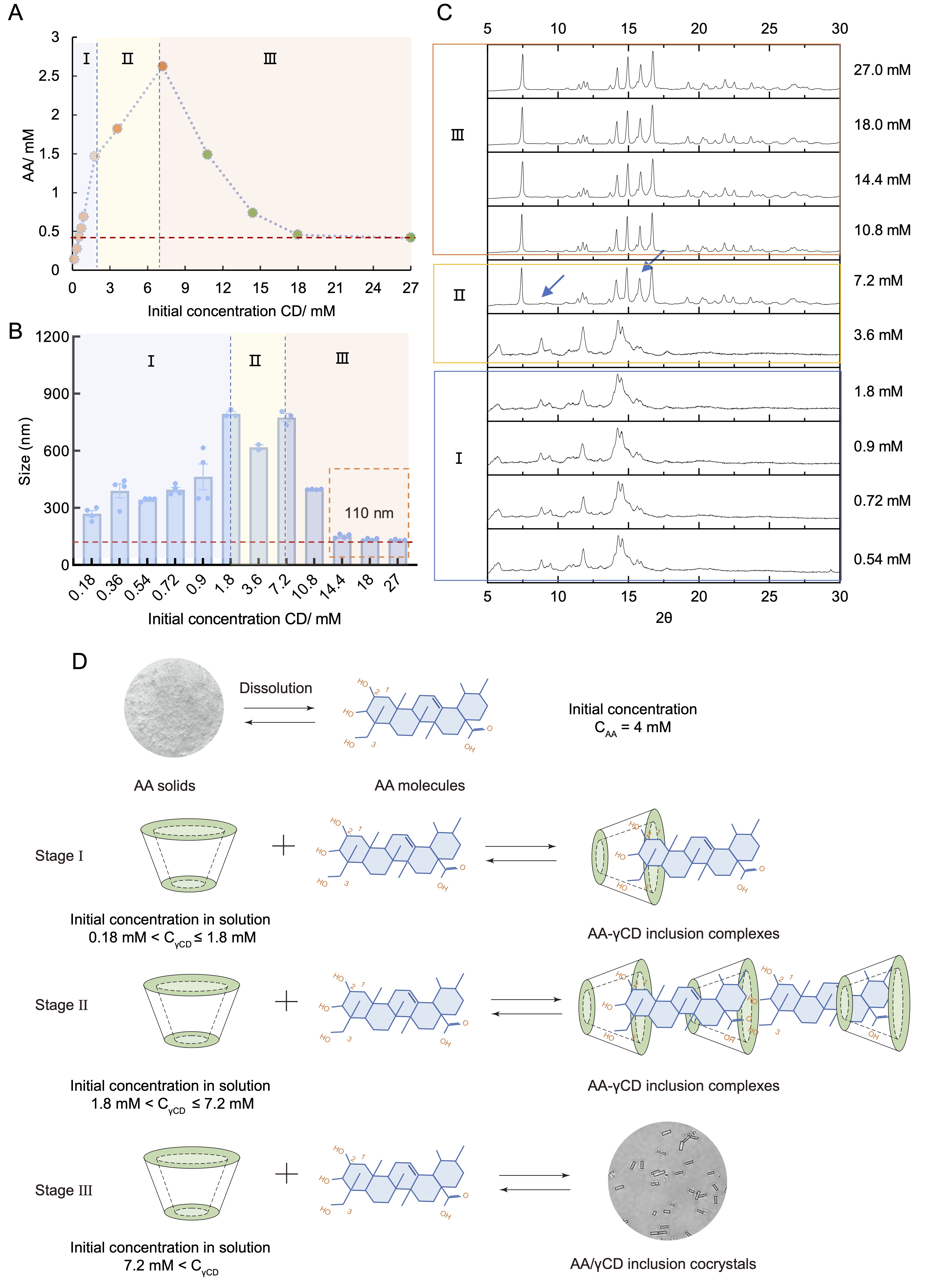
**

**Fig. S4** Phase solubility of AA in ascending concentration of γCD includes stage Ⅰ and stage Ⅱ.

**
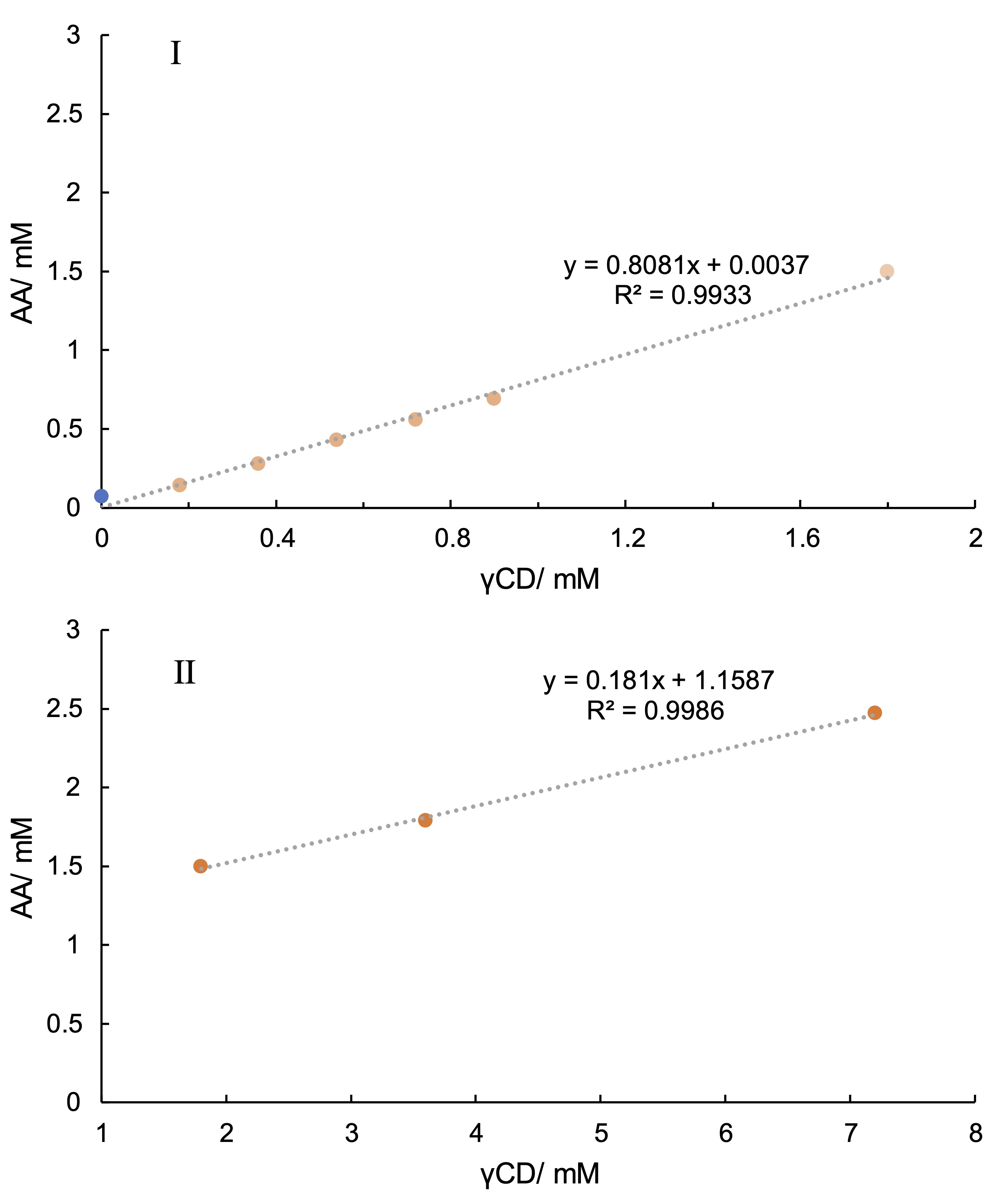
**

**Fig. S5** The concentration of γCD vs initial added concentration in solution.

**
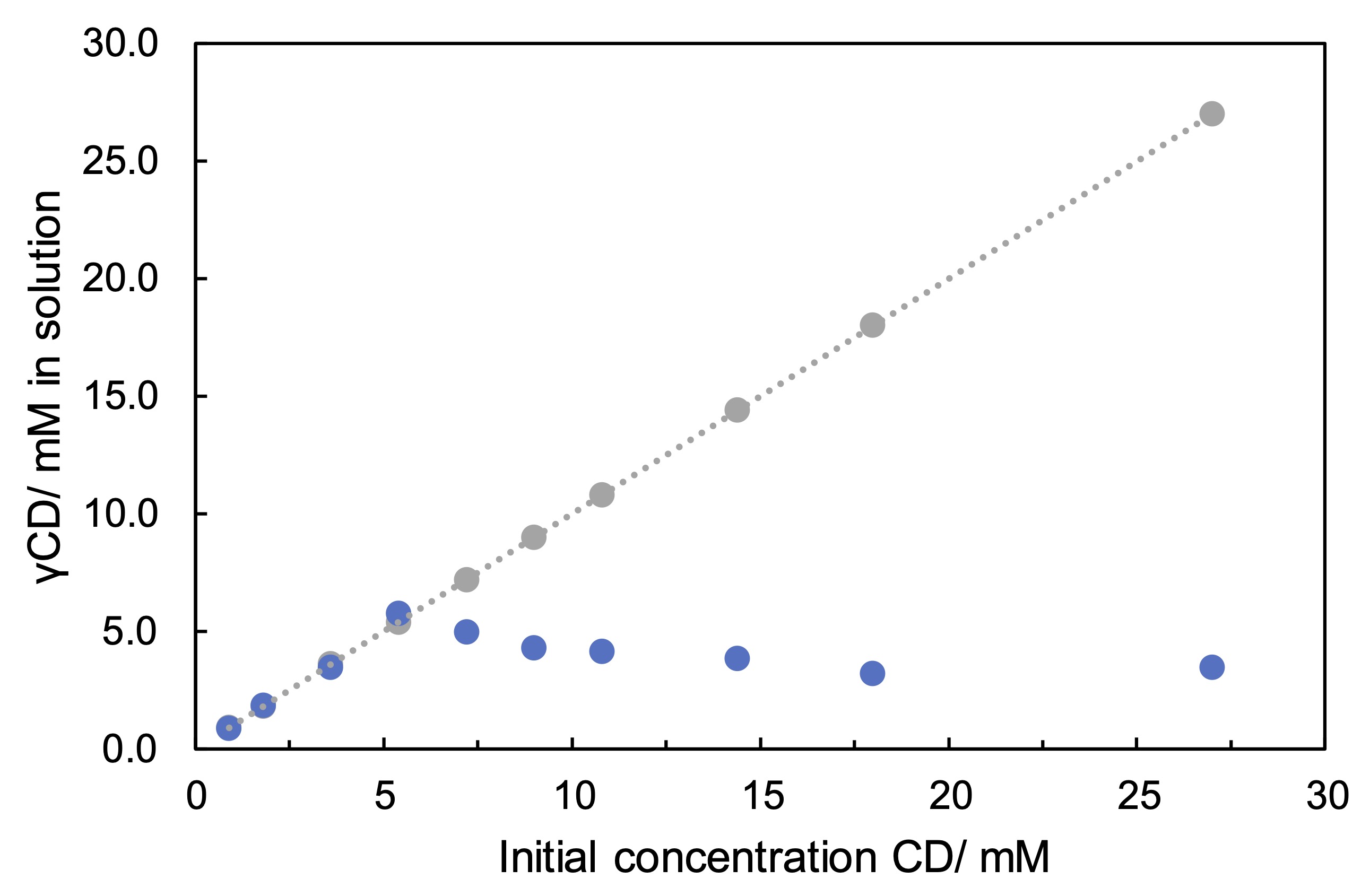
**

**Table S2**. Primer sequences for qPCR experiment

| Primer | Sequence |
| --- | --- |
| TNF-α F | CTCATGCACCACCATCAAGG |
| TNF-α R | ACCTGACCACTCTCCCTTTG |
| IL6 F | CTTCCATCCAGTTGCCTTCT |
| IL6 R | CTCCGACTTGTGAAGTGGTATAG |
| IL1α F | CCTTACACCTACCAGAGTGATTT |
| IL1α R | AACCAAGTGGTGCTGAGATAG |
| IL1β F | GCAGAGCACAAGCCTGTCTTCC |
| IL1β R | ACCTGTCTTGGCCGAGGACTAAG |
| iNOS F | GTTCTCAGCCCAACAATACAAGA |
| iNOS R | GTGGACGGGTCGATGTCAC |
| IL10 F | TTCTTTCAAACAAAGGACCAGC |
| IL10 R | GCAACCCAAGTAACCCTTAAAG |
| β-actin F | TGTCCACCTTCCAGCAGATGT |
| β-actin R | AGCTCAGTAACAGTCCGCCTAGA |
